# Measuring client trust in community health workers: A multi-country validation study

**DOI:** 10.7189/jogh.11.07009

**Published:** 2021-03-10

**Authors:** Pooja Sripad, Tracy L McClair, Alain Casseus, Sharif Hossain, Timothy Abuya, Ann Gottert

**Affiliations:** 1 Population Council, Washington, D.C., USA; 2 Zanmi Lasante, Mirebalais, Haiti; 3 Population Council, Dhaka, Bangladesh; 4 Population Council, Nairobi, Kenya

## Abstract

**Background:**

Client trust in community health workers (CHWs) is integral for improving quality and equity of community health systems globally. Despite its recognized conceptual and pragmatic importance across health areas, there are no quantitative measures of trust in the context of community health services. In this multi-country study, we aimed to develop and validate a scale that assesses trust in CHWs.

**Methods:**

To develop the scale, we used a consultative process to conceptualize and adapt items and domains from prior literature to the CHW context. Content validity and comprehension of scale items were validated through 10 focus group discussions with 75 community members in Haiti, and Kenya. We then conducted 1939 surveys with clients who interacted with CHWs recently in Bangladesh (n = 1017), Haiti (n = 616), and Kenya (n = 306). To analyze the 15 candidate scale items we conducted a split sample exploratory/confirmatory factor analysis (EFA/CFA), and then assessed internal consistency reliability of resulting set of items. Finally, we assessed convergent validity via multivariable models examining associations between final scale scores with theoretically related constructs.

**Results:**

Factor analyses resulted in a 10-item Trust in CHWs Scale with two factors (sub-scales): *Health care competence* (5 items) and *Respectful communication* (5 items). The qualitative data also underscored these two sub-domains. The full scale had good internal consistency reliability in Bangladesh, Haiti and Kenya (alphas 0.87, 0.86, and 0.92, respectively; all alphas for subscales were also > 0.7, most > 0.8). Greater scores on Trust in CHWs were positively associated with increased client empowerment, familiarity with CHWs, satisfaction with recent client-CHW interaction, and positive influence of CHW on client empowerment. Scale scores were not influenced by the age, sex, parity, education, and wealth quintiles in across countries and may be affected by contextual factors.

**Conclusions:**

The Trust in CHWs Scale, which includes *Health care competence* and *Respectful communication *sub-scales, is the first such scale developed and validated globally. Our findings suggest this 10-item scale is a reliable and valid tool for quantifying clients’ trust in CHWs, with potential utility for tracking and improving CHW and health systems performance over time.

Gauging clients’ trust in health workers is critical to evaluating health system performance and ultimately to ensuring equitable access to high-quality health services [1]. Within the health systems literature, trust is commonly recognized as an individual’s positive expectation that their needs will be adequately met (and that they will not be harmed) by a health provider or system. Attributes of trust within a client-provider relationship include a client’s implicit vulnerability, a provider’s fiduciary role, and underlying asymmetries in information and power [2-4]. In low- and middle-income countries (LMICs) with under-resourced health systems, clients’ trust in community health workers (CHWs) is particularly critical given CHWs are often the first point of contact for residents of low-income, rural, peri-urban or urban-informal settlements with limited access to facility-based care [5]. Given CHWs’ unique intermediary position as educators, counselors, and service providers within complex and mixed service delivery systems, trust in CHWs also becomes an essential outcome of CHW performance [6,7].

The phenomenon of trust in health systems broadly, and of CHWs, is garnering increasing attention in community health research and health systems accountability in LMICs. Qualitative studies demonstrate the relevance of client trust in health systems and resonance across service areas, including reproductive, maternal, newborn and child health (RMNCH), adolescent health, HIV/AIDS, and other infectious diseases [8-10]. In their exploration of trust, these studies have largely focused on interactions between clients and facility-based health care providers, with emerging themes around health care competence, including confidentiality, and those related to respectful communication. Studies investigating client-CHW interactions generally suggest client trust aligns closely with community acceptability of CHWs. While the “cultural and social familiarity” of CHWs within communities underlies trust, these studies suggest that client trust in CHWs may relate to how responsive the wider health system is to CHWs and clients, client preferences for male or female CHWs, how CHWs are paid, a CHW’s time spent living and engaging in the community, and a client’s satisfaction of provided services [11-13].

Despite recognition of the importance of trust in CHWs, as well as its conceptual maturity [14] in LMICs, there are no existing measures of trust in CHWs. Similar to qualitative research described earlier, existing trust scales focus mainly on client trust in facility-based providers or in health systems, in higher income countries [7,15-17]. Such measures assessing trust in health systems in high income settings can be adapted to CHW contexts in LMIC settings [18]. Given the varied scopes of work that CHWs implement globally as well as the health-area agnostic yet context-specific relevance of client trust in CHWs, it is critical for any composite scale to be tested in multiple LMICs. CHWs are lay workers with up to six months of initial training who provide care in community settings. In our study, CHWs include Family Welfare Assistants (FWAs) in Bangladesh, Agents de Santé Communautaire Polyvalents (ASCPs) in Haiti, and Community Health Volunteers (CHVs) in Kenya. While the FWAs are focused on family planning, ASCPs cover general health, and CHVs cover a range of services including MNCH. These cadres are government-supported but often supplemented by the NGO sector and allocated base monthly salaries/stipends of 9000-21 800 BDT (US$107-260), 12000 HTG (US$100), and 2500 KSH (US$25) in Bangladesh, Haiti, and Kenya respectively.

The purpose of this study was to develop and validate a Trust in Community Health Workers (CHWs) Scale from the perspective of CHWs’ clients that: (a) comprehensively reflects the construct of trust; (b) is practically relevant for improving CHW performance; (c) is sufficiently brief (readily integrated into surveys and/or monitoring activities); and (d) works across sociocultural geographies and health areas. This study is part of the Frontline Health project, that seeks to harmonize and propose transferrable measures to capture CHW performance and functionality within community health systems [7].

## Methods

### Study design and setting

This study applied a scale development approach [19], drawing on existing trust scales, qualitative work [9,17], and expert consultation to conceptualize trust in CHWs as embedded within a larger health system. The composite trust scale was then implemented and validated in client surveys conducted as a part of studies in Bangladesh, Haiti, and Kenya evaluating aspects of CHW performance. We applied a split sample exploratory/confirmatory factor analysis (EFA/CFA) in which half the sample in each country was used for EFA, and half for CFA, then assessed reliability, construct, and convergent validity. Finally, we explored associations between trust and wealth, education, and sub-geographies within each country.

The scale was tested in three countries in three geographically distinct regions with varied sociocultural, economic and political contexts and maturity of CHW programs. The study was conducted in 12 unions in Keraniganj sub-district in Bangladesh, three communes in the lower Artibonite and Centre departments in Haiti, and four sub-counties in Bungoma and Kilifi counties in Western and Coastal Kenya.

### Participant sample and method

The sample includes clients who interacted with a CHW recently: women who received family planning counseling from FWAs within the last 6 months (Bangladesh), women who received antenatal or postnatal counseling and care in the last 3 months (Kenya), and women and men who interacted with ASCPs in the last 6 months about any health issue (Haiti).

Data collectors were extensively trained and obtained informed consent from all participants by signature or thumb print for those with limited literacy. Data collectors administered client surveys in local languages (Bangla in Bangladesh, Creole in Haiti, and Kiswahili in Kenya), in client’s homes in Bangladesh and Haiti and in health facilities in Kenya. Surveys were paper-based in Bangladesh and tablet-based using Commcare and Open Data Kit software in Haiti and Kenya, respectively.

Ethical approval was obtained from the Population Council Institutional Review Board in New York USA (*p874*, *p879*, and *p876*), the Bangladesh Medical Research Council (*20608052019*), the Zanmi Lasante Institutional Review Board in Haiti (*ZLIRB2732019*), and AMREF Health Africa Ethics and Social Review Committee in Kenya (*p573*).

### Scale development process

We developed conceptual domains and a pool of testable items by reviewing prior literature, with emphasis on an earlier qualitative study of client trust in CHWs in Kenya [9,17]. These conceptual domains and items were then further adapted through a consultative process within the study team and selected items from The Health Care Relationship (HCR) Trust Scale, that focused on health promotion but maintained key elements that measured trust in primary health care (PHC) settings. Items from this scale were selected reflecting relevant conceptual domains identified in the earlier client-CHW trust study (eg, health care competence, confidence, communication, integrity, and systems trust). Conceptual domains and items were further refined with consideration for specific community health contexts in Bangladesh, Haiti, and Kenya, resulting in a set of 15 ‘candidate’ items, grouped under four hypothesized domains: health care competence, communication quality, integrity/caring demeanor, and respect for persons/equal treatment. In all three countries, after an introductory statement, “thinking back over interactions you’ve had with CHWs over the last 6 months,” respondents were asked how often CHWs had performed certain actions/exhibited certain qualities. Response options were: ‘never’, ‘some of the time’, ‘most of the time’, and ‘all of the time’.

To qualitatively assess face and content validity of the 15 ‘candidate’ items, we conducted two FGDs in Haiti with men (n = 6) and women (n = 7) selected purposively through Zanmi Lasante staff and CHW supervisors who had access to recent clients of CHWs. We first asked about what trust means to them in an open-ended format then, following self-administration of the potential items, asked a set of questions related to each item. We asked: ‘What does this statement mean to you?’, ‘Is this a useful/relevant statement?’, ‘Does the language make sense?’, ‘How can it be changed?’, ‘Should we keep or remove it?’. Respondents generally understood each question, found each to be relevant, and suggested the study team retain all 15 items. In Kenya, we conducted 10 FGDs with 62 purposively selected women in an open-ended format that asked what trust meant them to in relation to CHWs to further content validate the scale.

Prior to survey implementation in each country, the 15 translated items were checked for meaning and comprehension by the study team, and items were pretested with potential respondents to refine item wording and confirm translations of items.

### Analysis

Stata 13 software (Stata Corp, College Station, TX, USA) was used for statistical analysis. We explored response distributions for each item to confirm that each had adequate variation (eg, not > 90% in a category) to continue with psychometric analyses. We then randomly split each country’s study sample designating half for exploratory factor analysis (EFA) and half for confirmatory factor analysis (CFA). Trust questions were scored between 1 to 4 (‘never’ to ‘all of the time’).

Following recommended criteria [19], we began our EFA by conducting a principal component analysis (PCA) to explore the number of factors underlying the set of items by assessing the number of eigenvalues over 1.00, generating scree plots and conducting parallel analysis to gauge the number of factors above the ‘elbow’ (for scree plot) or line (for parallel analysis). Items with low uniqueness (< 0.7), low factor loadings (< 0.3), and were conceptually necessary and interpretable were retained for the CFA model.

We conducted a CFA using the second set of split samples across the three countries to test the emergent factor structure from the EFA. We retained items with statistically significant factor loadings (*P* < 0.05). Statistical tests measuring model adequacy were conducted and interpreted based on common criteria including the root mean square error of approximation (RMSEA) of < 0.10 (ideally < 0.05), comparative fit index (CFI) of > 0.90 (ideally > 0.95), Tucker-Lewis Index (TLI) f > 0.90 (ideally > 0.95), and the standardized root mean square residual (SRMR) of < 0.08. We integrated modification indices into the CFA’s structural equation model to adjust for similarly worded items and reassessed goodness-of-fit for the two sub-scales and the full two-factor scale.

Qualitative analysis of trust domains and item congruence was done in two stages. First, a rapid preliminary analysis of community responses in Haiti determined the pool of testable items; second, a thematic analysis in Haiti and Kenya confirmed the two-factor structure. Thematic analysis followed an iterative process of reading, categorizing, and applying a codebook to the transcripts using NVivo 12. Trust themes and sub-themes were triangulated with the scale items and factor domains.

For the final set of items, internal consistency reliability was assessed on the full sample for each country using standard correlation tests and related criteria. Reliability, or internal consistency of items, was assessed using Cronbach’s alpha and ordinal theta; the latter assumes polychoric correlation, useful when items have limited response categories (four in the case of assessed items) [20]. A standard reliability cutoff of 0.7 was adopted to assess adequate internal consistency, ie, how well the items relate to one another to capture the overall construct of Trust in CHWs. Finally, we assessed convergent validity by exploring associations between full scale scores, as well as sub-scale scores, with theoretically correlated constructs extent of client empowerment, interactions with CHWs, influence of CHW on client empowerment, and satisfaction of services from a CHW. Bivariate and multivariable regression models were constructed, controlling for age, education, wealth, sex (Haiti only), parity (Bangladesh only), and union/commune/sub-county. Across all countries, wealth quintiles, constructed using a principal components analyses of various household assets, were distributed at 20% across the study population [21]. The union/commune/sub-county spread included 12 unions in Bangladesh (Konda, Taghoria, Shuvadda, Sakta, Taranagar, Rohitpur, Kalatia, Hozratpur, Aganagar, Zingira, Basta, Kalindi); 3 communes in Haiti (Mirebalais, Verrettes, Petite Riviere Artibonite); and 4 sub-counties in Kenya (Kaloleni, Torgaren, Webuye West, Kilifi North). In Bangladesh and Kenya, clustered client responses with respect to a recent CHW visit were also accounted for in multivariable models assessing convergent validity. **Table 1** includes sociodemographic characteristics of the full samples by country.

**Table 1:** Sample characteristics by country

	Bangladesh (n = 1017)	Haiti (n = 616)	Kenya (n = 306)	
**% or mean/SD/range**	**% or mean/SD/range**	**% or mean/SD/range**	
**Sex:**			
-Female	100	85.1	100
-Male		14.9	
**Age in years**	24.6/5.1/15.0-35.0	39.0/17.9/15.0-90.0	26.5/6.1/16.0-45.0
**Parity**	1.8/0.9/0.0-5.0	n/a	n/a
**Highest education attended/completed:**			
-None	5.6	58.3	8.2
-Primary	31.9	25.5	53.3
-Secondary and above	62.5	16.2	38.6

SD – standard deviation

### Convergent validity measures

**Client Empowerment in Community Health Systems (CE-CHS)** was a validated 16-item Likert scale assessing respondents’ personal agency around health, agency in sharing health information with others, and engagement in community health systems (mean score, range: 1-4) [21].

**Frequency of interaction with CHWs** was captured categorically one time, two to three times, and 4 or more times, and captured interactions within the last 6 months in Haiti and Bangladesh and in the last 3 months in Kenya.

**Influence of CHW on client empowerment** was measured using Likert responses to a 5-item scale in Bangladesh and Kenya and a 2-item index in Haiti.

**High satisfaction with recent CHW visit/interaction** was measured converting a 4-point scale, which we collapsed into a binary measure that captured participant responses of being ‘very satisfied’ compared to all other categories.

Country-specific variation in the survey questions for convergent validity measures are described elsewhere [21]. Multivariable sub-analyses allowed us to explore associations between trust and socioeconomic status (wealth and education) and other demographics.

## RESULTS

A total of 1939 clients recently interacted with CHWs and completed surveys in Bangladesh (n = 1017), Haiti (n = 616) and Kenya (n = 306) (**Table 1**). All participants in Bangladesh and Kenya were female, and 85% were female in Haiti. Respondents in Bangladesh and Kenya were age 25 years on average; respondents in Haiti were age 40 years on average. Education levels varied with a high proportion of respondents with no education in Haiti (58%) compared to Bangladesh and Kenya (< 10%).

**Table 2** describes the final 10 Trust in CHWs Scale items including a breakdown of responses for each item, grouped under the two final sub-scales (see detail below): *Health care competence* and *Respectful communication*. Individual item responses across settings skewed positively toward “all of the time” or “most of the time”, with highest levels in Haiti.

**Table 2:** Trust in CHWs Scale item frequencies, summary scores, and factor loadings, by country

	Bangladesh (n = 1017)						Haiti (n = 616)									Kenya (n = 306)								
	**All of the time, %**	**Most of the time, %**	**Some of the time %**	**Never, %**		CFA* factor loading	**All of the time, %**	**Most of the time, %**		**Some of the time, %**		**Never, %**		CFA* factor loading		**All of the time, %**	**Most of the time, %**		**Some of the time %**		**Never, %**		CFA* factor loading	
**Trust in CHWs** Scale mean (SD)	3.49 (0.45)						3.79 (0.43)									3.39 (0.65)								
**Health care competence:**																								
1. How often have you felt the CHW was telling you everything you needed to know about your health-related problems?	64.41	25.07	8.16	2.36		*0.60*	92.05	1.95		4.06		1.95		*0.65*		50.98	27.12		16.99		4.90		*0.80*	
2. How often have you felt the CHW knew as much as s/he should about a health topic?	70.21	26.06	3.64	0.10		*0.66*	89.45	4.38		4.38		1.79		*0.86*		50.33	30.07		14.38		5.23		*0.78*	
3. How often has the CHW taken enough time with you during visits?	54.08	38.94	6.10	0.88		*0.77*	91.23	3.90		3.57		1.30		*0.78*		51.96	29.41		14.71		3.92		*0.68*	
4. How often has the CHW kept what you discussed confidential/private from others in your community?	54.57	26.94	15.24	3.24		*0.37*	85.67	2.61		3.91		7.82		*0.35*		67.32	15.03		8.17		9.48		*0.46*	
5. In your opinion, how often has the CHW been committed to providing the best care possible?	52.02	41.89	5.11	0.98		*0.74*	75.81	8.93		11.69		3.57		*0.32*		58.17	27.78		9.80		4.25		*0.75*	
Sub-scale mean (SD) and* higher order CFA loading*	3.48 (0.48)					*0.98*	3.75 (0.50)							*0.90*		3.32 (0.70)							*n/a*	
**Respectful communication:**																								
6. How often has the CHW been an excellent listener?	55.85	38.94	4.52	0.69		*0.69*	92.37		2.92		2.92		1.79		*0.45*	66.67		24.18		7.52		1.63		*0.73*
7. How often have you felt better (emotionally) after seeing/talking to the CHW?	50.44	40.61	8.46	0.49		*0.61*	86.53		4.71		6.33		2.44		*0.45*	44.44		31.05		14.05		10.46		*0.55*
8. How often has the CHW treated you with respect?	73.75	22.81	2.46	0.98		*0.61*	96.43		2.11		0.81		0.65		*0.65*	79.08		15.36		3.92		1.63		*0.80*
9. How often has the CHW had your best interest at heart?	54.77	37.27	7.18	0.79		*0.90*	95.45		2.44		1.14		0.97		*0.84*	68.95		20.92		7.19		2.94		*0.89*
10. How often has the CHW made you feel that you were worthy of his/her time and effort?	52.61	40.90	6.00	0.49		*0.81*	94.64	1.79		2.27		1.30		*0.84*		67.97		21.24		7.84		2.94		*0.85*
Sub-scale mean (SD) and* higher order CFA loading*	3.50 (0.49)					*0.88*	3.86 (0.41)							*0.59*		3.46 (0.64)								*n/a*

CFA – confirmatory factor analysis, CHW – community health worker

*All factor loadings were significant at the *P* < 0.001 level. Abbreviations include standard deviation (SD) and confirmatory factor analysis (CFA). CFA sample sizes were 508, 308, 153 in Bangladesh, Haiti, and Kenya respectively.

Split sample exploratory factor analyses explored the factor structure of the 15 initial items across Bangladesh (n = 509), Haiti (n = 308), and Kenya (n = 153). PCA, parallel analysis, and scree plots suggested two factors in Bangladesh and Haiti and one or two factors in Kenya. Given non-normally distributed item responses and correlated factors, we used the iterative principal factor specification and tested these plausible factor structures under an EFA with Promax rotation.

Within the EFA, three items were removed due to uniqueness greater than 0.7 (‘How often have you discussed options and choices with the CHW before making a health care decision?’, ‘How often has the CHW asked about what else was happening in your life?’, ‘How often have you felt comfortable talking to the CHW about what else was happening in your life?’). Interpretability of these items was likely mixed because item wording and variable norms of whether CHWs discussed non-health issues during their interactions with clients. Two items were dropped due to problematic wording (eg, ambiguous or double-barreled), as determined by investigators during analysis. One item with high uniqueness was retained because of conceptual importance of confidentiality to trust, per FGD findings (‘How often has the CHW kept what you discussed confidential/private from others in your community?’). The EFA identified two factors each comprised of five items, the first pertaining to perceptions of CHWs’ competence in health care provision and the other pertaining to perceptions of respect shown in communications with the respondent as a client/community member.

Using the CFA samples from Bangladesh (n = 508), Haiti (n = 308), and Kenya (n = 153), we conducted a CFA for the two sub-scales emerging from the EFA: *Health care competence* (5 items) and *Respectful communication* (5 items). The confidentiality/privacy item was tested in both factors given its mixed loadings in the EFA in different settings. All item factor loadings were adequate (< 0.3) for each subscale in each country; most were above 0.6. The two sub-scales were significantly correlated (*P* < 0.001) in Bangladesh (0.70); Haiti (0.79) and Kenya (0.85).

We also conducted a higher-order CFA, with the hypothesized structure in which the two factors load on a higher-order factor (trust in CHWs). This model converged in Bangladesh and Haiti but not in Kenya. A unidimensional scale of all 10 items may fit better in Kenya, as suggested by the high correlation between sub-scales (eg, sub-factors less conceptually distinct). Robust and significant factor loadings in the CFA affirmed and validated the Trust in CHWs Scale (10 items) (**Table 2**).

After reviewing modification fit indices, we added several correlated error terms that should be added to the model. Goodness of fit statistics (**Table 3**) was acceptable, with RMSEAs < 0.1, CFIs and TLIs > 0.9 (in some cases > 0.95), and SRMR< 0.05. Statistics for the higher order model are not presented for Kenya since (as noted) this model did not converge.

**Table 3:** Model-fit statistics – confirmatory factor analysis (CFA), by country*

	Bangladesh	Haiti	Kenya
Trust in CHWs Scale (10 items):			
-RMSEA	0.074	0.039	n/a
-CFI	0.960	0.988	n/a
-TLI	0.942	0.980	n/a
-SRMR	0.035	0.030	n/a
No. correlated *error* terms added	3	7	
**Sub-scales**			
*Health care competence* (5 items):			
-RMSEA	0.048	0.061	0.048
-CFI	0.991	0.985	0.993
-TLI	0.981	0.969	0.986
-SRMR	0.020	0.029	0.021
No. correlated error terms added	0	0	0
*Respectful communication* (5 items):			
-RMSEA	0.060	0.070	0.073
-CFI	0.995	0.988	0.990
-TLI	0.982	0.971	0.979
-SRMR	0.013	0.018	0.027
No. correlated error terms added	2	1	0

CHW – community health worker, RMSEA - root mean square error of approximation, CFI – comparative fit index, TLI – Tucker-Lewis Index, SRMR – standardized root mean square residual

*CFA sample sizes were 508, 308, 153 in Bangladesh, Haiti, and Kenya respectively.

### Reliability

Internal consistency reliability, assessed using Cronbach’s alpha and Ordinal theta, were all above the 0.7 threshold in each country (**Table 4**). The Trust in CHWs Scale exhibited a cross-sample internal consistency of 0.86-0.92 (alpha) and 0.93-0.96 (theta). Sub-scales *Health care competence* and *Respectful communication* also demonstrated high internal consistency (alpha = 0.73-0.85/theta = 0.84-0.91; alpha = 0.85-0.88/theta = 0.91-0.94; respectively).

**Table 4:** Reliability of Trust in CHWs Scale and sub-scales, by country

	Bangladesh (n = 1017)		Haiti (n = 616)		Kenya (n = 308)	
	Alpha	**Ordinal theta**	**Alpha**	**Ordinal theta**	**Alpha**	**Ordinal theta**
**Trust in CHWs Scale** (10 items)	0.87	0.93	0.86	0.96	0.92	0.96
**Sub-scales:**						
*Health care competence* (5 items)	0.73	0.84	0.76	0.85	0.84	0.91
*Respectful communication* (5 items)	0.85	0.91	0.85	0.91	0.86	0.94

CHW – community health worker

Mean scores for the Trust in CHWs Scale were 3.49, 3.79, and 3.39 in Bangladesh, Haiti, and Kenya respectively. Mean scores for the *Health care competence* sub-scale were 3.48, 3.75, and 3.32 and the *Respectful communication* scale were 3.5, 3.86, and 3.46 in Bangladesh, Haiti, and Kenya, respectively. Extent of dispersion was similar across the three countries and Haiti shows the highest levels of Trust in CHWs as well as in the sub-scales.

### Construct validity

Qualitative data from the FGDs in Haiti and Kenya confirmed that the set of trust items resonated with participants’ sense of their community’s closeness with and dependability on CHWs as accountable and equitable health intermediaries. Respondents focused on two main aspects of trust that support the two-factor psychometric solution – *Health care competence* and *Respectful communication*. In contrast to the three initially hypothesized factors, interpersonal communication and respect appear to fall within a single phenomenon (**Table 5**). Respondents agreed that trust in CHWs is instrumental in promoting their own health and well-being. In Haiti, while qualitative data suggested possible gendered interpretations of trust in CHWs – women tended to associate trust with secrecy, confidentiality, and communication and men with reliability, competence, and integrity, quantitative data revealed no difference in overall conceptualization by sex (next section). Though equitable/fair treatment is embedded in trust definitions in both countries, in Kenya, levels may vary by client and CHW backgrounds (eg, higher trust if clients and CHWs are from “the same clan”). In both settings, narratives associate trust in CHWs with familiarity and frequency of interaction, services provided/used and/or referrals, accompaniment to and advocacy at facilities, and continued follow up over time.

**Table 5:** Construct validity: supporting quotations

	Haiti	Kenya
**Trust in CHWs**	*“CHWs are very trusted…We are guaranteed help – if we have problems, we can call them to speak. In addition, most of them have something called professional secret (confidentiality)… CHWs are trained people… People are comfortable with their CHW.”* (Women, Haiti)	*“By the way they tell us, listen and are willing to help despite their inability to not help. They offer solutions to most of our concerns. Sometimes it is better for someone to give you advice that will help in the long run than give you food that helps only at a particular moment.”* (Women, Kenya)
**Health care competence**	*“I trust [CHWs] because they know what they are doing; they are trained to do it... For instance, if someone feels sick around about 2AM and we have an agent’s number, they can guide us on what we can do for the person in the meantime.”* (Women, Haiti)	*“She is like a community doctor that is why we trust her.”* (Women, Kenya)
	*“We can trust them [CHWs] by telling them anything…and [they] do not speak a word of it*.” (Women, Haiti)	*“I will trust her because for example if I have a problem that I cannot tell anyone else, I will go to her and tell her; she can keep it confidential and she can attend to me well…she can get the medicine for me secretly and people will not know.”* (Women, Kenya)
**Respectful communication**	*“[The CHW] is always available and willing to listen to people’s problems. This means when you are explaining a problem, they take time to listen and give you the advice you need.”* (Men, Haiti)	*“I normally feel they are not biased*.” (Women, Kenya)
		*“She [CHW] becomes concerned and follows up even after I have gone to the hospital. She will talk and explain to me stuff as she passes by.”* (Women, Kenya)

CHW – community health worker

### Convergent validity

We assessed convergent validity by examining multivariable associations, controlling for sociodemographic characteristics, between the Trust in CHWs Scale scores and conceptually related constructs (**Table 6**) and related sub-analyses. The frequency of CHW interactions varied by country, though the majority across all three settings reported interacting more than once. Haiti’s sample most frequently interacted (four or more visits) compared to Bangladesh and Kenya (63% vs 21%) (**Table 6**). The mean CE-CHS score on a 4-point scale was 2.38, 2.79, and 3.00 in Bangladesh, Haiti, and Kenya, respectively. Mean score for CHW influence on empowerment was 2.19 and 3.18 in Bangladesh and Kenya, respectively (range: 0 to 4) and 0.91 in Haiti (range: 0-1). Satisfaction with CHW services varied by country: 32%, 93%, and 47% were very satisfied in Bangladesh, Haiti, and Kenya, respectively.

**Table 6:** Distribution of convergent validity items by country

	Bangladesh (n = 1017)	Haiti (n = 616)	Kenya (n = 306)
**% or mean/SD/range**	**% or mean/SD/range**	**% or mean/SD/range**
**Empowerment**	2.30/0.38/1.04-4.00	2.79/0.39/1.00-4.00	3.00/0.41/1.76-4.00
Frequency of interaction with a CHW:*†			
-One time	22.42	11.04	30.70
-Two to three times	56.74	25.81	49.70
-Four or more times	20.85	63.15	19.60
Influence of CHW on empowerment	2.19/0.56/1.00-4.00	0.91/0.25/0.00-1.00	3.18/0.52/1.00-4.00
Highly satisfied with recent CHW visit	32.45	92.86	47.06

CHW – community health worker, SD – standard deviation

*In the last 6 months (Bangladesh and Haiti); in the last 3 months or since childbirth (Kenya).

†Missing 10 observations in Kenya.

Multivariable models showed that all three countries (**Table 7**) controlled for age, education, wealth, union/commune/sub-county, sex (Haiti only), and parity (Bangladesh only). These country-specific models showed that the Trust in CHWs Scale was positively and significantly associated with the CE-CHS Scale in Bangladesh and Haiti (at *P* < 0.01 and *P* < 0.001 levels, respectively), but did not show an association in Kenya. The Trust in CHWs Scale was positively and significantly associated (mostly at *P* < 0.001) with more frequent interactions with a CHW, satisfaction with recent CHW interaction, and positive CHW influence on empowerment. Sub-scales associations showed similar, robust associations.

**Table 7:** Associations between scales and related constructs*

	Bangladesh (n = 1017)			Haiti (n = 616)			Kenya (n = 306)		
	**Trust in CHWs**	*Health care competence*	***Respectful communication***	**Trust in CHWs**	***Health care competence***	***Respectful communication***	**Trust in CHWs**	***Health care competence***	***Respectful communication***
Empowerment	0.10¶ (-0.01, 0.21)	0.17‡ (0.07, 0.28)	0.03 (-0.10, 0.15)	0.33§ (0.24, 0.41)	0.32§ (0.22, 0.43)	0.33§ (0.25, 0.41)	0.09 (-0.07, 0.27)	0.12 (-0.06, 0.30)	0.08 (-0.10-0.26)
Frequency of interaction with CHW (ref: one visit):									
Two to three times	0.14† (0.02, 0.25)	0.19‡ (0.07, 0.30)	0.09 (-0.04, 0.21)	0.14† (0.02, 0.26)	0.18† (0.03, 0.32)	0.10¶ (-0.02, 0.21)	0.30‡ (0.09, 0.51)	0.34‡ (0.12, 0.57)	0.25† (0.06,0.46)
Four or more times	0.27§ (-0.15, 0.40)	0.32§ (0.19, 0.45)	0.23‡ (0.09, 0.37)	0.26§ (0.15, 0.37)	0.29§ (0.16, 0.42)	0.23§ (0.12, 0.33)	0.56§ (0.33, 0.78)	0.63§ (0.39, 0.87)	0.49§ (0.26, 0.71)
Influence of CHW on empowerment	0.19§ (0.10, 0.28)	0.21§ (0.12, 0.29)	0.17‡ (0.07, 0.28)	0.85§ (0.73, 0.98)	0.89§ (0.74, 1.04)	0.83§ (0.70, 0.95)	0.46§ (0.27, 0.65)	0.52§ (0.33, 0.70)	0.40§ (0.19, 0.61)
Very satisfied with recent CHW visit	0.26§ (0.18, 0.33)	0.24§ (0.16, 0.31)	0.29§ (0.20, 0.37)	0.63§ (0.51, 0.75)	0.73§ (0.59, 0.88)	0.53§ (0.40, 0.64)	0.29§ (0.14, 0.45)	0.32§ (0.15, 0.49)	0.26‡ (0.11, 0.42)

*All values presented are beta coefficients. 95% Confidence Intervals are presented in parenthesis. Multivariable models control for: age, education, wealth, and union/commune/sub-county, sex (Haiti only), and parity (Bangladesh only). Not shown: no significant associations between Trust in CHWs Scale and sub-scales with age, education, wealth, sex (Haiti only), and parity (Bangladesh only).

†*P* < 0.05.

‡*P* < 0.01.

§*P* < 0.001.

¶*P* < 0.1

Sub-analysis of socioeconomic and demographic associations with Trust in CHWs showed no significant differences across country settings (data not shown). Multivariable models with covariates age, sex (Haiti), parity (Bangladesh), education, wealth (quintiles), and union/commune/sub-county and accounting for clustering by the CHW found no significant differences in trust by age, sex, parity, education, and wealth. They did show some variability in the Trust in CHWs Scale by sub-geography; clients reported higher levels of trust in 6 of the 12 unions in Bangladesh (βs = 0.22-0.51, *P* < 0.001) and lower levels of trust in one sub-county in Kenya (β = -0.66, *P* < 0.001). There were no significant differences in trust across communes in Haiti.

## DISCUSSION

In this study, we developed and validated the Trust in CHWs Scale. The scale is comprised of two sub-scales, *Health care competence* (5 items) and *Respectful communication* (5 items). The full scale and sub-scales demonstrated strong factor loadings, goodness-of-fit, reliability, and convergent validity. While scale scores were uninfluenced by age, sex, parity, education, and wealth quintiles across countries, our findings suggest the client-CHW trust relationship may be affected by contextual factors, including CHW programming and support. We recommend using the Trust in CHWs Scale (**Table 8**) in future research and health systems strengthening efforts, as a valid, comprehensive, programmatically relevant, cross-context-applicable measure of clients’ trust in CHWs.

**Table 8:** Trust in community health workers (CHWs) Scale and Scoring

Think about ALL of the interactions you’ve had with CHWs in the last 6 monhts or so. Tell me how often you felt this way by responding ‘never’, ‘some of the time’, ‘most of the time’, ‘all of the time’*
**Health care competence:**
1. How often have you felt the CHW was telling you everything you needed to know about your health-related problems?
2. How often have you felt the CHW knew as much as s/he should about a health topic?
3. How often has the CHW taken enough time with you during visits?
4. How often has the CHW kept what you discussed confidential/private from others in your community?
5. In your opinion, how often has the CHW been committed to providing the best care possible?
**Respectful communication:**
6. How often has the CHW been an excellent listener?
7. How often have you felt better (emotionally) after seeing/talking to the CHW?
8. How often has the CHW treated you with respect?
9. How often has the CHW had your best interest at heart?
10. How often has the CHW made you feel that you were worthy of his/her time and effort?

*Scoring the Trust in CHWs Scale. Responses are scored as follows: 1 (never), 2 (some of the time), 3 (most of the time), 4 (all of the time). Generate mean sub-scale and full-scale scores by taking the mean of non-missing items for a final range of 1 to 4.

The two-factor solution aligns with qualitative research around client-CHW trust and community acceptance of CHWs more broadly [9,22]. As items fell out of the factor analyses, our four hypothesized domains transformed into two, with confidentiality (originally folded within integrity) incorporated into *Health care competence*, and communication quality and respect for persons/equal treatment folding into a single *Respectful communication*. Triangulation with the qualitative data from FGDs confirmed together these two sub-scales thematically captured trust as a single phenomenon. While we recommend using the full Trust in CHW Scale in future research and program monitoring, given application of both sub-scales to inform programming, robust sub-scales psychometrics and qualitatively confirmed content validity suggest each can also be independently integrated into surveys and program monitoring activities. Use of the full scale may be particularly important in certain settings that have less psychometric distinction between the two sub-scales, as was evident in Kenya.

Convergent validity findings demonstrate expectedly that increases in an individual’s trust in CHWs is positively associated with more frequent interactions with CHWs, influence of CHWs on empowerment, feeling very satisfied with a CHW’s visit, and in some cases (in Haiti and Bangladesh), one’s empowerment in the community health system. We recommend applying the Trust in CHWs Scale in future research related to contextual factors, client awareness of CHW payments (formal and informal), and in relation to empowerment. Contextual factors including social, cultural norms, values, beliefs and practices at the community level, as well as social class (of a CHW or client) may affect client-CHW relationships with implications for trust [23]. Payments, continuity of care, and client’s choice of provider have been shown in facility-based settings but underexplored in CHW contexts [24,25]. Irrespective of intended stipends for CHWs in Bangladesh (highest), Haiti (middle), and Kenya (lowest), their actual payment and support structure likely affects their motivation, commitment, and consequently influences their behaviors toward client with downstream implications for trust in CHWs. Clients’ level of empowerment for following CHW advice may influence a positive feedback loop on trust as a dynamic construct [9]. The cross-sectional client surveys prevented our assessing predictive validity of the scale with outcomes of interest that reflect this dynamism. Future research should apply longitudinal approaches to investigate associations with client behavior (eg, service use/follow up, referral completion, or return to the CHW for future problems) as well as health outcomes.

Findings also suggest that the Trust in CHWs Scale can be applied across different geographic and cultural settings, different CHW scopes of practice (eg, generalized vs focused), and variably financially-compensated CHWs– although it will be important to continue testing the scale across additional contexts. These findings are consistent with literature around trust in health care workers and health systems that also suggests such commonalities in the conceptualization of trust [18]. While we found trust to be internally consistent and externally convergent when controlling for individual characteristics (eg, age, sex, parity, education, wealth), levels of Trust in CHWs varied across countries. Trust levels were similar in Kenya and Bangladesh and very high in Haiti – the latter having the highest frequency of client-CHW interactions and the least politically and economically stable context. This aligns with research emphasizing that interpersonal trust in health care workers and impersonal trust in health institutions builds over time [9,17,24] and also that CHWs play integral roles in humanitarian settings like Haiti, as primary sources of care and advice [26,27]. Confounding by sub-geographies in Kenya and Bangladesh suggests that contextual, historical, and program factors may influence Trust in CHWs. Unlike findings in high-income countries documenting lack of trust in PHC providers as socioeconomic and racial disparities increase [28], the Trust in CHWs Scale showed no association with education and wealth across settings. This may be due to the fact CHWs in our study settings tend to be of similar socioeconomic status and race/ethnicity as the clients they serve.

The Trust in CHWs Scale can be used in combination with other metrics [21,29] to measure and improve CHW performance and provide insight into how community-integrated health systems are functioning through special studies or routine data collection as recommended by Agarwal and colleagues [7]. It could measure effects of policy and program strategies set forth by the World Health Organization’s CHW Guidelines and Community Health Roadmap priorities, as well as complement the inter-agency derived Minimum Quality Standards and Indicators for Community Engagement [30-32]. National surveys can integrate this short set of 10 items into routine monitoring for quality improvement and accountability mechanisms for community health. Given that Trust in CHWs is a client’s perception of how responsive CHWs are and also a proxy for upholding social contract of the wider health system as a social institution [2], we recommend civil society and service implementers adopt it in program assessments and reporting for accountability.

Our findings should be interpreted alongside the study’s limitations. One limitation was that we were unable to conduct FGDs with women in Bangladesh to content validate the scale; however, qualitative data from CHWs (data not shown) within the study scope show the resonance of Trust in CHWs Scale in this setting. Another limitation was Kenya’s relatively small sample size, which precluded disaggregation of women receiving antenatal vs postnatal care from CHWs. We were unable to test for invariance by sex in Haiti given that there were only 44 males in the CFA sample; though we maintained power within the full sample to assess no difference in trust levels by sex. Future studies should ensure sufficient power to test for variation by notable sub-group characteristics such as sex, urban/rural residence, socioeconomic status, age, and minority status. Finally, our results may not be generalizable to other populations within each country, to other countries, or to other CHW health focus areas. Indeed, this is a critical avenue for future research.

## CONCLUSION

We developed the Trust in CHWs Scale – the first such scale validated globally – a promising multi-dimensional measure that can contribute to monitoring and improving CHW performance, as well as community health program functioning and accountability more broadly. We recommend further investigation of the transferability of this scale globally, for example in national surveys, monitoring activities, and other community health research.
